# Effects of Attrition Shoes on Kinematics and Kinetics of Lower Limb Joints During Walking

**DOI:** 10.3389/fbioe.2022.824297

**Published:** 2022-02-09

**Authors:** Shane Fei Chen, Yan Wang, Yinghu Peng, Ming Zhang

**Affiliations:** ^1^ Department of Biomedical Engineering, Faculty of Engineering, The Hong Kong Polytechnic University, Hong Kong, Hong Kong SAR, China; ^2^ Hong Kong Polytechnic University Shenzhen Research Institute, Shenzhen, China

**Keywords:** attrition shoes, lower extremity, multibody calculation, motion analysis, joint biomechanics

## Abstract

Shoe attrition is inevitable as wearing time increases, which may produce diverse influences on kinematics and kinetics of lower limb joints. Excessive attrition may change support alignment and lead to deleterious impacts on the joints. The study identifies the biomechanical influences of aging shoes on lower limb joints. The shoes in the experiment were manually worn in the lateral heel. Nineteen healthy participants, including thirteen males and six females, were recruited to conduct walking experiments wearing attrition shoes (AS) and new shoes (NS) with a random order. A Vicon motion analysis system was used to collect kinematic data and ground reaction force. Kinematic and kinetic parameters of the hip, knee, and ankle joints were calculated using the Anybody Musculoskeletal Model and compared between the two conditions, AS and NS. The results showed that wearing an attrition shoe decreased the plantarflexion angle and plantarflexion moment of the ankle joint, while significantly increasing the magnitude of the first peak of the knee adduction moment and hip abduction moment and hip internal rotation moment (*p* < .05). The results of the study implied that wearing attrition shoes is not recommended for those people with knee problems due to increase in medial loading.

## Introduction

Footwear is designed to protect humans from injuries in different environments (Barton et al., 2009) and provide assistance in motion control and attenuation of impact forces in daily activities. Shoes affect kinematics with a limitation of eversion and inversion range of motion of lower limb joints during walking (Morio et al., 2009; Sun et al., 2020; Dempster et al., 2021). However, aging attrition shoes (Moghaddam et al., 2019) change the plantar support surface and may affect the kinematics and kinetics of the lower limb, which might be related to joint injuries (Taunton et al., 2003; Ramsey et al., 2019). Compared with new shoes, worn shoes with decreasing cushioning capability lead to an increase of stance time and kinematic adaptational changes (Kong et al., 2009). Worn shoes were also demonstrated to increase energy cost and risk of injuries (Ramsey et al., 2018) and decrease lower limb stability (Saito et al., 2007). Footwear was found to reduce the external loading rate to protect lower limbs from injuries (Altman and Davis, 2016) and designed to reduce the knee adduction moment (Fisher et al., 2007; Peng et al., 2020) to relieve medial knee pain. Lateral-wedged insoles or shoes with a certain degree of inclination to the medial were normally adopted to be the interventions to decrease the first peak knee adduction moment that was beneficial to patients with medial knee osteoarthritis (Radzimski et al., 2012; Felson et al., 2019; Sinclair and Stainton, 2019).

Patterns of shoe attrition could be classified into lateral, medial, and central degradation (Baumfeld et al., 2015). Lateral heel degradation was found to be the most popular pattern according to a measurement of over 200 shoes, with an average shoe age of almost a year (Sole et al., 2014). In an assessment of 76 participants (Finestone et al., 2012), most of the outsole abrasion presented a posterolateral pattern with an average of 12° and 5 cm^3^ abrasion volume. There is lack of studies to understand the biomechanical effects of shoe abrasion on lower limb biomechanics.

This study aimed to identify the immediate effects of worn shoes on kinematics and kinetics of the lower limb. As the most popular worn pattern, lateral heel attrition with mediolateral asymmetry was set as the experimental condition. It is hypothesized that lateral heel attrition would increase the knee adduction moments.

## Methods

### Participants

Nineteen participants were recruited to participate in the study. This sample size was estimated using G*power 3.0.10, Universität Düsseldorf, Germany, and the minimum sample size was 15 with a significant level of 0.05 and a statistical level of 0.8. The medium effect size of 0.8 was adopted in the sample size calculation. The average age of participants was 25 ± 5 years. The means and standard deviations of body mass and height were 69.5 ± 12.2 kg and 173.6 ± 9.3 cm, respectively. All participants had no histories of lower extremity injuries during the experiments. This study was approved by the Human Participants Ethics Sub-Committee of The Hong Kong Polytechnic University (Number: HSEARS20150121003). Informed consent was obtained from all participants before experiments.

### Footwear Conditions

**FIGURE 1:**
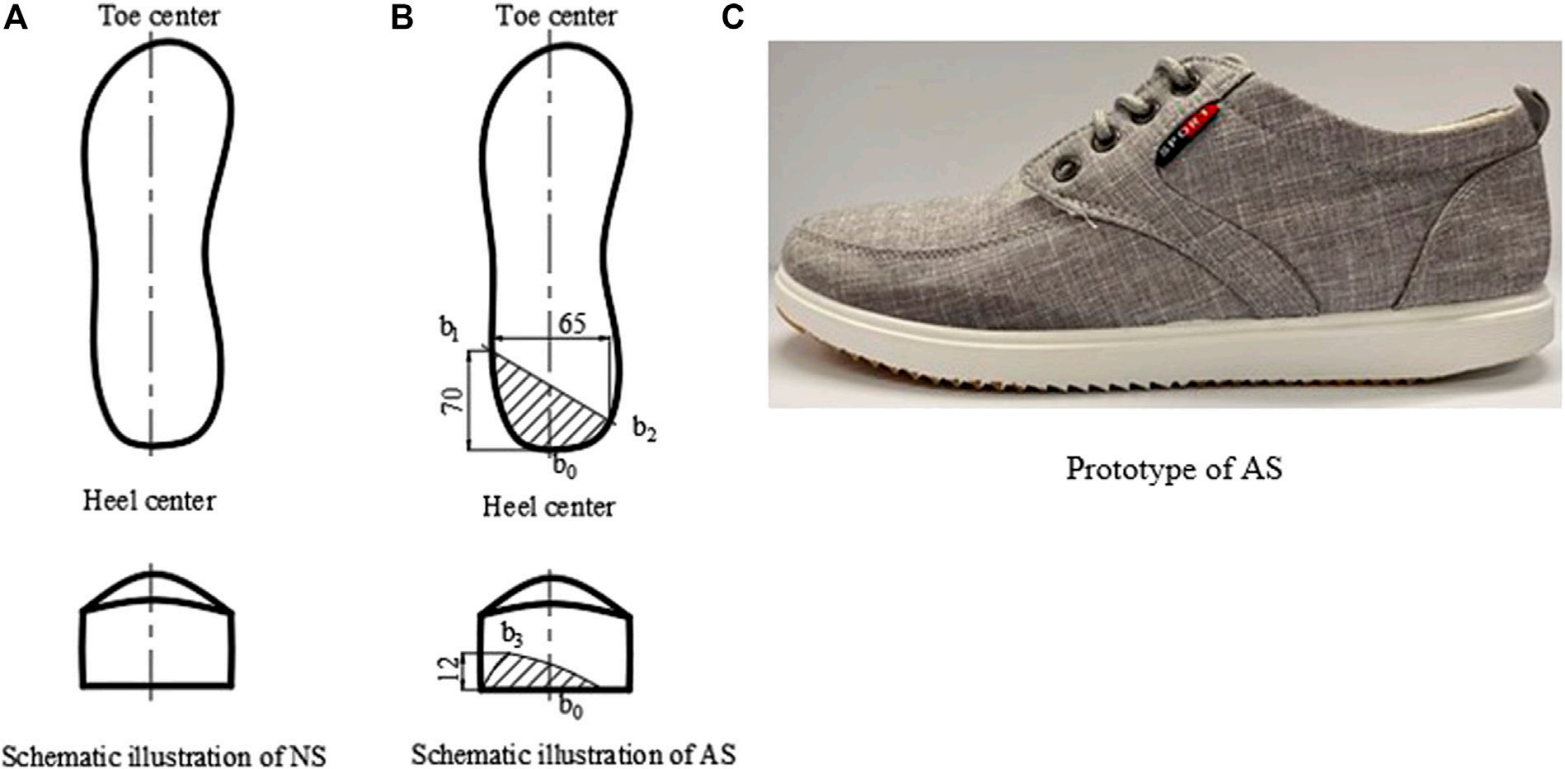
Scheme of shoe conditions. **(A)** Schematic illustration of NS. **(B)** Schematic illustration of AS. **(C)** Prototype of AS.

Canvas shoes were adopted for the preparation of non-attrition (new shoes, NS) and attrition shoes (attrition shoes, AS). Attrition shoes (AS) with lateral worn heels were adopted, which is the most popular attrition pattern (Finestone et al., 2012; Sole et al., 2014). As shown in Figure 1B, the degree of attrition was measured by the length of two lines, where one is the line connecting the lateral attrition point $b_{1}$ and heel center $b_{0}$ and the other is the line connecting the lateral attrition point $b_{1}$ and the other attrition point $b_{2}$ . The maximum attrition position is at point $b_{3}$ in the lateral heel portion, as shown in Figure 1B. A prototype of AS is shown in Figure 1C.

### Experiment Setup and Procedure

A Vicon motion capture system with eight cameras (Vicon, Oxford Metrics Ltd., Oxford, England) was used to collect motion data at 150 Hz, and two force platforms (OR6, AMTI, Watertown, United States) were used to measure the ground reaction force (GRF) at 1000 Hz. The participants wore tight-fitting clothes, and reflective markers were attached on bony landmarks to define the lower limb into seven segments. The marker set followed a previous study (Peng et al., 2020). The participants were required to walk for a few minutes to accommodate each pair of shoes in random order. They were required to walk at their natural walking speed during the walking trials. Each participant completed three successful trials with each condition of shoes.

### Musculoskeletal Model

The lower limb musculoskeletal (MSK) model using the Anybody Modeling System (version 6.0.3, AnyBody Technology, Aalborg, Denmark) was used in this study. The generic MSK model was based on the anthropometric database of the Twente Lower Extremity Model (TLEM 1.1) (Klein Horsman et al., 2007). The experimental data provided input for the MSK model to calculate the kinematics and kinetics of the lower extremity joints (Peng et al., 2018). The marker trajectory data and anthropometrics for each participant were used to optimize the MSK model, and then, kinematic parameters of the lower limb joints were calculated. After the kinematical calculation, inverse dynamic calculation was conducted to obtain kinetics of the joints.

### Statistical Analysis

SPSS (Version 22.0, IBM, Chicago, IL, United States) was used to perform statistical analysis. Peak values of angles and moments of ankle, knee, and pelvis joints and vertical GRF were extracted for statistical analysis of the two conditions. The joint moments were normalized with body weight and body height (BW and·BH) (Peng et al., 2020). Mean values of all variables were calculated from three successful trials of each shoe condition for each participant. The data distribution was testified to be normal when the Shapiro–Wilk test with significance level 0.05 was performed. Post hoc paired t-tests with Benjamini–Hochberg adjustment (P^*^) were conducted to determine the relationship between the two conditions with a significance level of 0.05.

## Results

**FIGURE 2:**
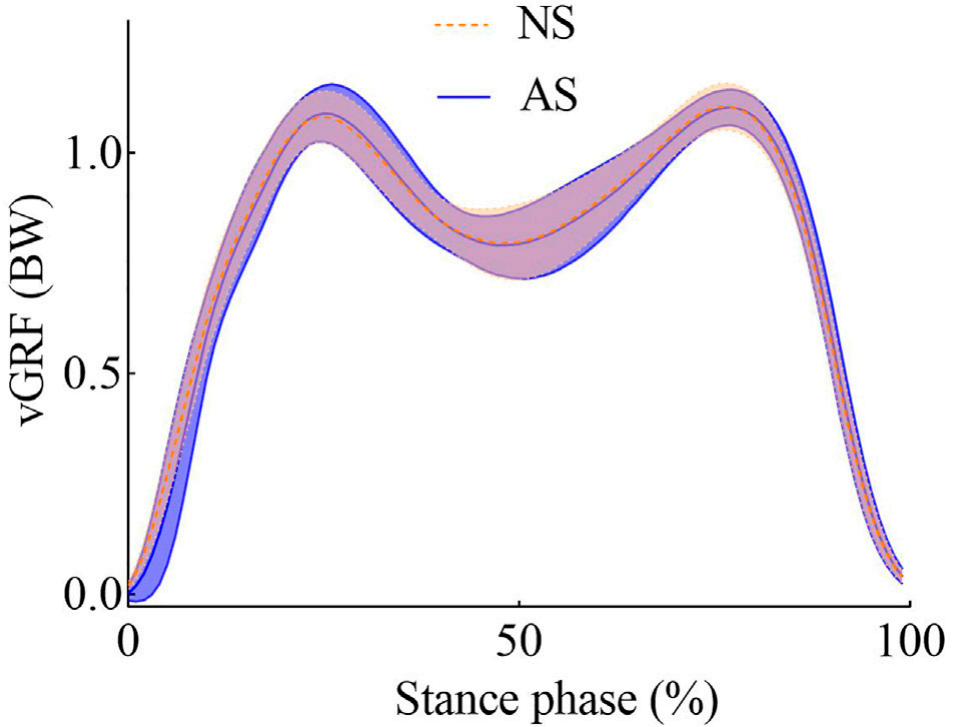
Vertical GRF for NS and AS during stance.

Figure 2. shows the mean vertical GRF during the stance phase. The vertical GRFs were normalized by the body weight. Mean vertical GRF for different shoe conditions was calculated and showed no statistical difference with *p* > .05.

**FIGURE 3:**
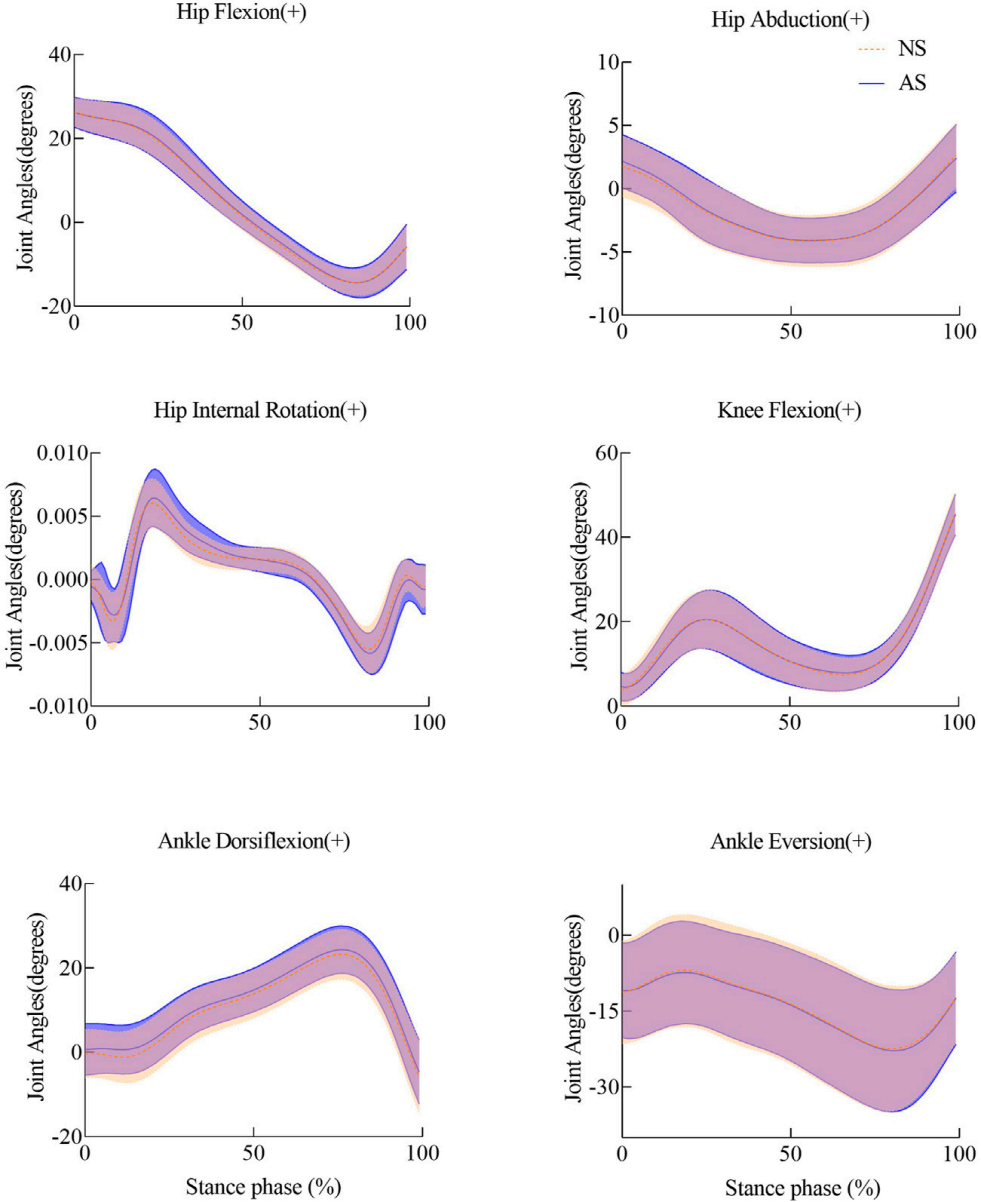
Joint angles of lower extremities with shade ± 1 std for NS and AS during the stance phase.

The mean and standard deviation (SD) of peak angles of lower extremity joints for the two conditions for all participants are listed in Table 1. Joint angles of lower extremities showed no significant difference during the stance phase shown in Figure 3.

**TABLE 1:** Peak angles of lower limb joints for NS and AS during stance (°).

Parameter	NS mean (SD)	AS mean (SD)	*p-*values	P^*^
Hip				
flexion	26.199 (3.639)	26.270 (3.672)	0.761	0.027
extension	−14.587 (3.246)	−14.611 (3.509)	0.923	0.041
external	14.284 (4.211)	14.313 (4.330)	0.947	0.050
rotation
internal rotation	4.708 (6.133)	5.283 (6.782)	0.141	0.014
abduction	3.199 (2.468)	3.235 (2.326)	0.822	0.032
adduction	−4.384 (2.091)	−4.372 (1.861)	0.946	0.045
Knee				
flexion	45.461 (5.358)	45.357 (4.665)	0.864	0.036
Ankle				
eversion	−5.213 (11.201)	−6.053 (10.003)	0.230	0.018
inversion	−23.019 (11.700)	−23.415 (11.626)	0.578	0.023
dorsiflexion	24.499 (2.534)	25.651 (3.339)	0.057	0.009
plantarflexion	−2.466 (2.506)	−0.828 (3.336)	0.027	0.005

Significant difference at *p-*value < P^*^ when walking with AS was compared with walking with NS.

**FIGURE 4:**
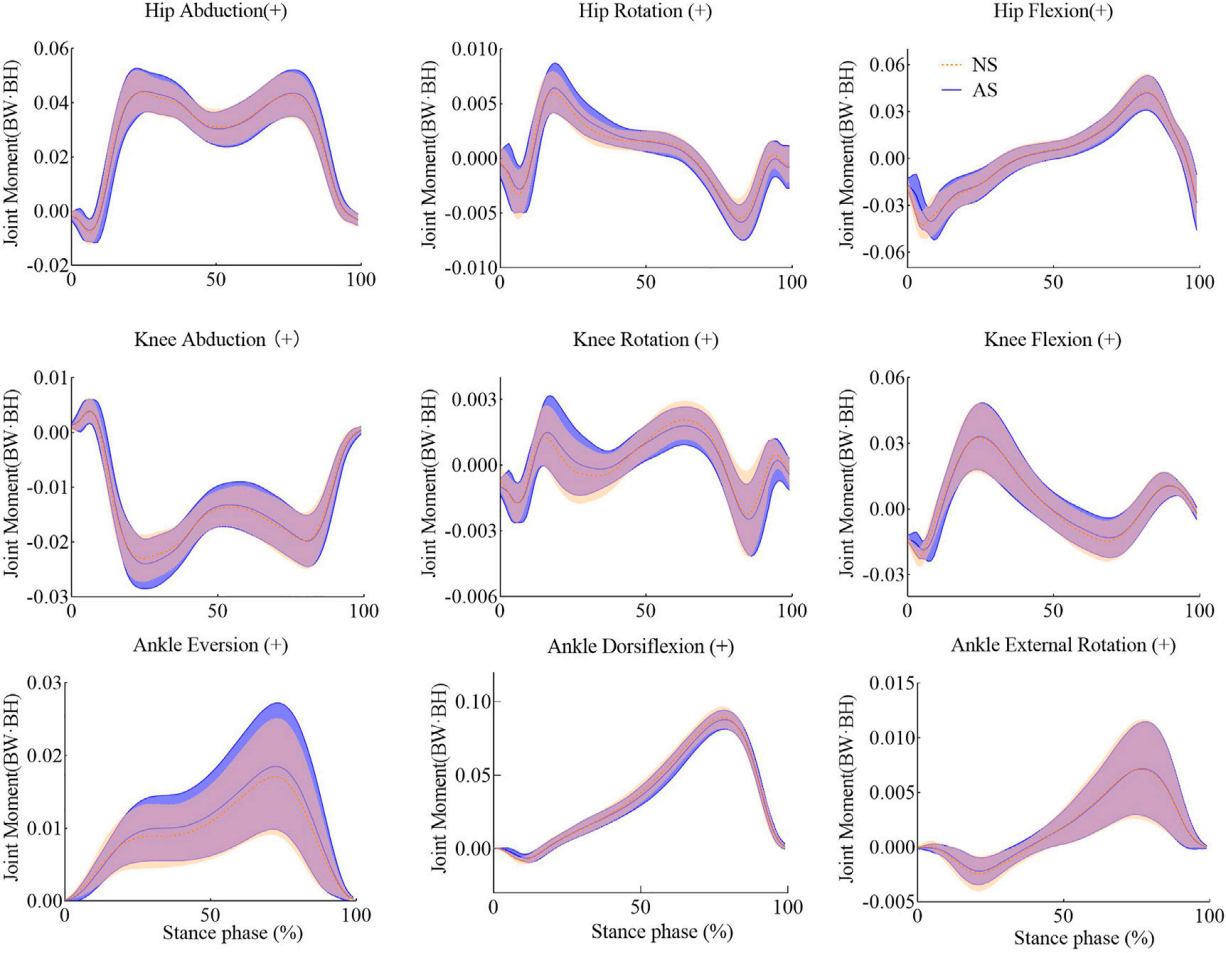
Joint moments of lower extremities with shade ± 1 std for NS and AS during the stance phase.

The joint moments were normalized by dividing the body weight and body height (BW and·BH) of each participant. Details of joint moments are shown in Table 2. The amplitudes of the hip internal rotation moment and the second hip abduction moment increased significantly in the AS group. The first peak of the knee adduction moment for the AS group revealed an increase of 8.3% compared with that of the NS group. The peak moments of the ankle joint showed a slight decrease. Joint moments during the stance phase are shown in Figure 4.

**TABLE 2:** Peak moments of lower limb joints for NS and AS during stance (BW and·BH).

Parameter	NS mean (SD)	AS mean (SD)	*p-*value	P^*^
Hip				
flexion rotation	0.044 (0.012)	0.043 (0.012)	0.443	0.039
extension rotation	−0.035 (0.012)	−0.038 (0.014)	0.130	0.022
external rotation	0.006 (0.002)	0.007 (0.002)	0.062	0.017
internal rotation	−0.005 (0.002)	−0.006 (0.002)	0.000^1^	0.003
first abduction	0.046 (0.008)	0.047 (0.008)	0.049	0.014
second abduction	0.043 (0.008)	0.045 (0.008)	0.004^1^	0.006
Knee				
Flexion	0.033 (0.016)	0.034 (0.015)	0.286	0.033
extension	−0.015 (0.009)	−0.014 (0.009)	0.064	0.019
external rotation	0.001 (0.001)	0.001 (0.001)	0.982	0.050
internal rotation	−0.002 (0.001)	−0.002 (0.001)	0.186	0.028
first adduction	−0.024 (0.004)	−0.026 (0.004)	0.006^1^	0.008
second adduction	−0.020 (0.006)	−0.021 (0.005)	0.310	0.036
Ankle				
eversion	0.018 (0.007)	0.019 (0.008)	0.173	0.025
inversion	−0.005 (0.001)	−0.005 (0.001)	0.816	0.044
external rotation	0.003 (0.001)	0.003 (0.001)	0.452	0.042
internal rotation	−0.008 (0.002)	−0.008 (0.002)	0.926	0.047
dorsiflexion	0.090 (0.008)	0.089 (0.007)	0.233	0.031
plantarflexion	−0.008 (0.002)	−0.007 (0.003)	0.011^1^	0.011

1 Significant difference at *p-*value < P^*^ when walking with AS was compared with walking with NS.

## Discussion

In this study, the effects of attrition shoes on lower limb joints were revealed by comparing them with those of new shoes during walking. Joint angles showed no significant effects, but some of joint moments of the lower limb displayed significant changes. The amplitudes of the ankle plantarflexion moment decrease significantly in the AS group as compared to those of the NS group. However, the amplitudes of the second hip abduction moment and internal rotation moment increased significantly when participants wore attrition shoes. Meanwhile, the first peak of the knee adduction moment revealed a significant increase of 8.3% when the AS group was compared with the NS group.

For the ankle joint, though the amplitude of the plantarflexion angle showed no significant effects, it tends to decrease while walking in worn shoes. Meanwhile, the amplitude of the plantarflexion moment showed a slightly significant decrease. AS lack one wedge part in the lateral heel, which prolongs the onset of heel strike. Thus, it leads to a shortening time for the plantarflexion. The duration of the plantarflexion is decreased, which makes the foot dorsiflexion quick. However, during the loading phase, the plantarflexion of the ankle joint provides eccentric contraction for the tibialis anterior muscle (Jafarnezhadgero et al., 2020). The reduction of the plantarflexion would cause the tibialis anterior muscle to provide less contraction force, which results in larger landing impact. The lateral heel attrition shifts the contact center anteriorly, which reduces the lever arm that lowers. The reduction of the lever led to decrease of the peak of the plantarflexion moment as shown in Figure 4. Insufficient plantarflexion moment may generate a more flattening longitudinal arch (Kirby, 2017), which would lead to an increase of tensional force in the plantar fascia that has been reported to be one of the causes of ankle pain (Kogler et al., 1996). Furthermore, the plantarflexion moment plays an important role in counterbalance (Kirby, 2017). Previous studies have indicated that plantarflexors regulate whole-body angular momentum in the sagittal plane during balance control (Neptune and Mcgowan, 2011), and fallers created a decreased plantarflexion moment compared to non-fallers (Barak et al., 2006). Consequently, the lack of plantarflexion moment in the AS group may also cause problems in balance.

Kinematic parameters of knee joints show no significant differences between the NS group and AS group. However, the first peak of the knee adduction moment increases by 8.3% with a significant level of *p* = .006, as shown in Table 2 and Figure 4. Since the attrition concentrates on the lateral heel, there is no significant effect in the second peak of the knee adduction moment. Wedge soles are often adopted as interventions for knee osteoarthritis (OA) (Desmyttere et al., 2018). Although the attrition outsole in this study is not exactly like wedge outsoles, the heel of AS displays the same appearance with medial wedges to a certain degree. The increase of the amplitude of the knee adduction moment agrees with that found in the previous studies (Lewinson et al., 2013; Peng et al., 2020), which illustrates that the peak knee adduction moment increases as the wedge develops from lateral to medial. On the contrary, lateral wedges are beneficial for participants with medial knee OA patients because they can reduce the peak of the knee adduction moment; the AS is disagreeable for them as it increases the knee adduction moment. Higher knee adduction moment has also been reported to have a relationship with the development of knee pain (Mai and Willwacher, 2019). Consequently, the aging shoes are also not suitable for participants with knee pain.

Aging shoes make an impact on the kinetics of the hip joint. For the AS group, the peak moments of the hip internal rotation and abduction increase significantly when compared with the NS group as illustrated in Table 2 and Figure 4. Aging shoes were found to lower activities of the vastus medialis and rectus femoris muscles and strengthen the activity of the gluteus medius muscle during the loading phase while walking (Jafarnezhadgero et al., 2020). The activities of these muscles may lead to increase of hip internal rotation and abduction moments. Furthermore, the increase of the hip internal rotation moment may cause abnormal hip motions (Weidow et al., 2006), which may change the rotational alignment of other segments in the lower limb.

A few limitations in the study should be discussed. The present study examines the immediate effect of shoe degradation on lower limb joints. However, the wear of shoes was a persistent behavior, of which impact is gradual. Canvas shoes with flat outsoles were adopted in this study, but other types of shoes were not explored. The lateral heel of shoes was worn mechanically, which may have slight differences with naturally worn shoes. The sample size in the study was another limitation. The generalizability of the findings should be noted. Additionally, further research should identify the gender difference in shoe attrition. The effects of other attrition patterns on the lower limb joints require further research.

## Conclusion

Lateral heel attrition in outsoles is the most common degeneration pattern of shoes. The attrition shoes have significant impacts on the kinematics and kinetics of the lower limb joints. The peak plantarflexion angle and moment of the ankle joint decrease, and the first knee adduction moment and hip internal and abduction moments increase during walking for the AS group. Our findings in this study imply that aging shoes are not desirable, especially for those people with knee problems. Attrition in the heel also raises balance risk. Although the exact abrasion amount in aging shoes is not demonstrated for a shoe change, attrition shoes influence the walking pattern and result in discomfort.

## Data Availability

The raw data supporting the conclusion of this article will be made available by the authors, without undue reservation.
